# Beyond sequence similarity: toward function-based screening of nucleic acid synthesis

**DOI:** 10.3389/fbioe.2026.1832724

**Published:** 2026-05-14

**Authors:** Gary R. Abel, Tessa Alexanian, Craig Bartling, Jacob Beal, Samuel Curtis, Kevin Flyangolts, Leonard Foner, Samuel P. Forry, Gene D. Godbold, Eric Horvitz, Bin Hu, Corey M. Hudson, Caitlin Jagla, Rassin Lababidi, Sheng Lin-Gibson, Brittany Rife Magalis, Jaspreet Pannu, Sebastian Rivera, David Ross, Bruce J. Wittmann, James Diggans

**Affiliations:** 1 Fourth Eon Bio, San Diego, CA, United States; 2 Johns Hopkins University Center for Health Security, Baltimore, MD, United States; 3 International Biosecurity and Biosafety Initiative for Science, Geneva, Switzerland; 4 Battelle Memorial Institute, Columbus, OH, United States; 5 RTX BBN Technologies, Cambridge, MA, United States; 6 Center for AI Standards and Innovation (CAISI), National Institute of Standards and Technology, Washington, DC, United States; 7 Aclid, New York, NY, United States; 8 SecureDNA, Basel, Switzerland; 9 Biosystems and Biomaterials Division, National Institute of Standards and Technology, Gaithersburg, MD, United States; 10 Signature Science LLC, Charlottesville, VA, United States; 11 Microsoft, Office of the Chief Scientific Officer, Redmond, WA, United States; 12 Bioscience Division, Los Alamos National Laboratory, Los Alamos, NM, United States; 13 The Align Foundation, Covina, CA, United States; 14 Department of Biochemistry and Molecular Genetics, University of Louisville, Louisville, KY, United States; 15 Engineering Biology Research Consortium, Emeryville, CA, United States; 16 Twist Bioscience, South San Francisco, CA, United States; 17 International Gene Synthesis Consortium, Emeryville, CA, United States

**Keywords:** biological foundation models, biosecurity, DNA synthesis screening, function-based screening, protein function prediction

## Abstract

Synthetic nucleic acids are a key input to modern biotechnology, yet they represent dual-use materials that require robust screening to mitigate biosecurity risks. The prevailing screening paradigm, which identifies sequences of concern (SoCs) through sequence similarity to controlled pathogens and toxins, may not fully capture risks posed by AI tools that can decouple biomolecular function from reliance on known sequences. Rapidly advancing biodesign capabilities enable the generation of genes and proteins that might evade sequence-based detection. We highlight the critical need for function-based screening approaches that can detect sequences capable of hazardous biological functions, regardless of similarity to known SoCs. We examine the feasibility of function-based screening with an initial focus on proteins, arguing that, while protein sequence space is vast, biologically functional proteins are significantly constrained by biophysical and biochemical requirements that can be learned and modeled. We propose a concrete implementation framework organized along a continuum of complexity, starting with toxins as the most tractable targets before expanding to more complex pathogenic functions. We then discuss open challenges and describe a research and development strategy to address them.

## Introduction

Synthetic nucleic acids are a key input for a wide range of applications in biomedicine, biotechnology, and synthetic biology. Because synthetic biology research is fundamentally dual-use, nucleic acid synthesis screening serves as a critical biosecurity safeguard against both accidents and deliberate misuse (Mackelprang et al., 2025). Screening systems are meant to operate as rule-out tests, seeking to confirm with high confidence that an ordered sequence does not pose a biosecurity risk. The prevailing screening paradigm approximates this by comparing sequence similarity against known sequences of concern (SoCs), which are defined primarily by taxonomic origin of the source organism. If an ordered sequence is a “Best Match” to a known SoC when compared to a comprehensive database, the sequence is flagged; if not, it can be cleared (IGSC, 2024; UK DSIT, 2024; US NSTC, 2024). In practice they operate as limited rule-out tests for known SoCs and close variants. Customer screening serves as an independent safeguard against deliberate misuse, but is not a substitute for sequence screening.

While sequence-based screening has served as a foundation of biosecurity, its limitations are becoming increasingly relevant as both artificial intelligence (AI) and synthetic biology continue to advance. Challenges include unnecessary flagging of benign sequences from regulated organisms (Godbold et al., 2025b) and, more critically, failure to detect hazardous sequences that lack significant similarity to known SoCs, whether from unregulated organisms (Gemler et al., 2024; Williams et al., 2025), extensively modified or *de novo* designed proteins (Baker and Church, 2024; Wittmann et al., 2025; Hunter, 2024), or deliberate sequence obfuscation (Rose et al., 2024).

This is not a hypothetical future concern: AI-enabled protein design tools can already generate functional protein sequences that diverge substantially from natural sequences (Munsamy et al., 2024; Ruffolo et al., 2024; Sumida et al., 2024; Yeh et al., 2023). A redesigned toxin that binds the same cellular target as a natural toxin may have low sequence identity with any previously characterized protein. To current screening systems, such a sequence may appear novel^1^ and unremarkable (Wittmann et al., 2025), eroding the efficacy of sequence-based screening.

There is growing recognition that synthesis screening must move beyond definitions of SoCs based on taxonomic origin or sequence similarity alone, toward detecting sequences that encode hazardous biological functions (Mackelprang et al., 2025; Godbold et al., 2025b; Pálya and Nelson, 2026). Recent policy guidance from the United States (The White House, 2025; Vought and Kratsios, 2025), United Kingdom (UK DSIT, 2024), and European Union (EU DG SANTE, 2025) further reinforce this necessity.

We focus on two objectives. First, we examine the theoretical feasibility of function-based screening, arguing that fundamental biophysical and biochemical requirements constrain functional proteins and lead to learnable patterns that enable prediction of biomolecular properties from sequence.^2^ Second, we propose a concrete, near-term implementation framework that can begin to provide function-based screening capabilities while broader, more generalized predictive methods continue to mature. We contend that these two objectives represent points along a single developmental continuum, from targeted detection of specific known hazards toward increasingly general prediction of hazardous functions. Work on the nearer-term approach lays scientific and institutional foundations for the longer-term vision.

## Sequence-based and function-based screening are complementary

We define sequence-based screening as detection based on significant sequence similarity to a known SoC, i.e., a regulated gene or protein sequence. Current sequence-based screening methods employ techniques such as sequence alignment (Altschul et al., 1990), exact matching of cryptographically hashed k-mers (Baum et al., 2026), Hidden Markov Models (HMM) (Finn et al., 2011), k-mer signatures (Wyschogrod et al., 2022), and combinations thereof (Laird et al., 2025; Balaji et al., 2022; Gemler et al., 2023; Wheeler, Carter, et al., 2024).

**FIGURE 1:**
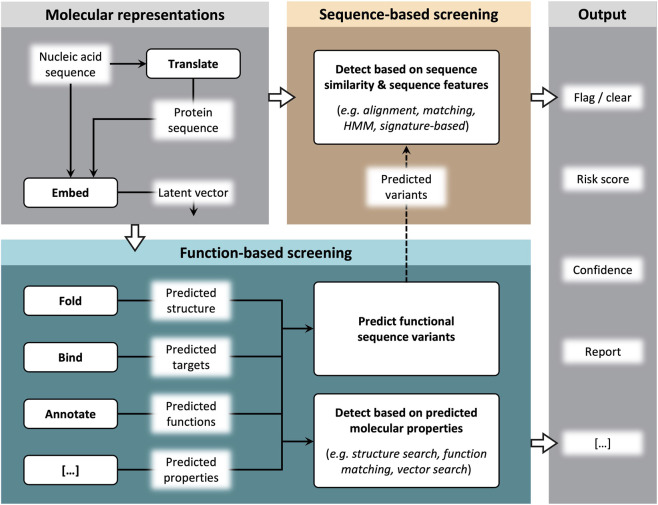
Conceptual overview of sequence-based and function-based screening elements. The molecular representations used as inputs to screening could include DNA and RNA sequences, translated protein sequences, and latent-space vectors from model embedding. Sequence-based screening detects sequences of concern through sequence similarity and sequence features (e.g., alignment, matching, HMM, or signature-based methods). Function-based screening utilizes computational methods to predict molecular properties such as structure, binding targets, and functional annotations, and detects functions of concern based on those predicted properties. New functional sequence variants can also be predicted and used to expand databases for sequence-based screening (dashed line). Both paradigms can produce a range of screening outputs that inform synthesis decisions. Note that elements shown are illustrative, not exhaustive.

In contrast, we define function-based screening as detection of sequences whose predicted molecular properties indicate a capacity for biological *functions of concern*, i.e., functions that contribute substantially to host toxicity or pathogenesis. Existing capabilities can be leveraged to implement function-based screening (Figure 1), including functional annotation (Lin et al., 2024), structure prediction (Meng et al., 2025) and search (van Kempen et al., 2024), binding prediction (Passaro et al., 2025), functional signature detection (Beal and Bryan, 2024), embedding space search (Pantolini et al., 2024), and prediction of functional variants to proactively expand sequence databases (Baum et al., 2026).

These terms are imperfect, as sequence-based methods implicitly capture some functional information, while function-based methods typically take sequences as input. Importantly, sequence-based and function-based screening need not be mutually exclusive. Indeed, some existing screening tools already incorporate elements of function prediction (Aclid, 2025; Balaji et al., 2022; Baum et al., 2026; Beal and Bryan, 2024; Gemler et al., 2023), and many methods fall along a spectrum between the two. The most effective screening approaches will likely combine elements from both paradigms by using hybrid approaches that integrate new methods into existing screening pipelines, enabling a smooth transition as models mature.

## Constraints on functional proteins enable prediction

The sequence space of all possible proteins is vast. For a protein of even a modest length of 75 residues, the number of possible sequences is far greater than the estimated number of atoms in the observable universe (Zhang et al., 2025). Yet the subregions corresponding to *biologically functional* proteins are constrained, with a much smaller subset representing hazardous functions. These constraints reflect fundamental requirements that a protein must satisfy to function successfully within one or more biological contexts, and further, to contribute to pathogenicity or toxicity. Biophysical constraints govern whether a sequence can fold into a stable, functional conformation or adopt a functional disordered ensemble, while biochemical constraints further restrict viable sequences, as specific molecular functions require precise spatial arrangement of catalytic residues and geometric complementarity at binding interfaces. These constraints have measurable consequences, and even a simple binding function may have fewer than one in 10^11^ functional sequences (Keefe and Szostak, 2001). The functional regions of sequence space are thus *many* orders of magnitude smaller and, crucially, are structured by a common set of biophysical and biochemical principles. The same constraints that make functional sequences rare also make them predictable.

**FIGURE 2:**
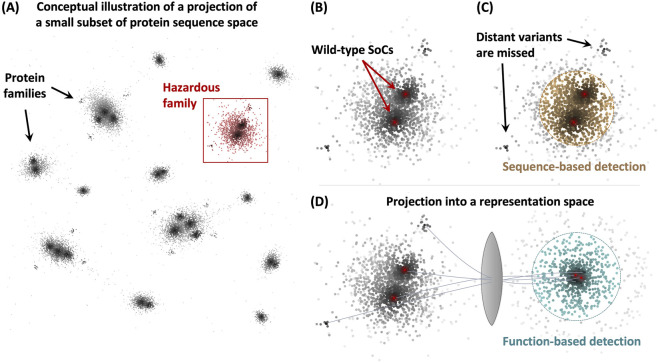
Conceptual illustration of a projection of a small subset of protein sequence space onto a two-dimensional grid. Each point represents a possible protein, with the point intensity corresponding to its activity for a particular function. (Not actual data). **(A)** Several isolated clusters corresponding to individual protein functional families are shown, with a specific hazardous family highlighted. **(B)** A close-up view of the hazardous cluster from (A), with two wild-type reference proteins shown as red dots surrounded by mutants of varying activity. **(C)** Current Sequence-Based Screening flags a subset of adjacent proteins, but misses any functional proteins that are sufficiently far away in sequence space (as indicated by arrows). **(D)** Function-based screening aims to project proteins into a different representation space where they cluster based on similarity in structure, function, and other molecular properties, to enable detection of functional analogs that may be distant in sequence space.

The constraints on functional proteins create statistical regularities in how sequence maps to structure and function, which can be learned empirically by AI models and exploited to detect specific functions of concern (Figure 2). In particular, biological foundation models have demonstrated a capacity for extracting such patterns and using them to predict protein properties (Bjerregaard et al., 2025; Li et al., 2024). There is growing evidence that foundation models can implicitly capture abstract representations of the underlying constraints on proteins (Ahdritz et al., 2024; Hayes et al., 2025; Brixi et al., 2025; Airas and Zhang, 2026), suggesting they might be able to recognize functional patterns in non-natural sequences through their latent space geometry. In the long term, such generalizable property prediction could unlock a more resilient approach to function-based screening, for example, by integrating across scales and modalities as an AI virtual cell (Bunne et al., 2024) to enable prediction of whether a novel protein would disrupt critical host cell processes.

It is not clear how far in the future generalized screening can be achieved, as the extent of such model capability generalization remains an open empirical question (Prabakaran and Bromberg, 2026). In the near term, however, the goal is more targeted: to identify sequences whose properties indicate specific harmful functions. This is a narrower target that can be approached incrementally, starting from targeted detection of specific known hazards and progressing towards broader function prediction as models mature.

## From theoretical feasibility to practical implementation

Function-based screening is both necessary and theoretically feasible. The practical challenge is to move toward operational deployment with limited data and imperfect models.

A useful starting point is to observe that synthesis screening does not require comprehensive sequence-to-function prediction. Instead, it only needs to prevent the acquisition of sequences that could do harm in the hands of a malicious or careless actor. Comprehensive function prediction asks “What does this protein do?”—a classification problem across a vast and poorly defined label space (Bileschi et al., 2022). Screening asks a narrower question: “Can we confidently exclude that this protein performs specific harmful function X?” where X is drawn from a known set of harmful functions. This is a binary exclusion problem for a set of narrow, well-defined targets.

This framing suggests a pragmatic near-term approach: before developing generalist models for broad function prediction, specialist models can be trained to detect one (or a few) specific functions of concern. For example, a specialist model for detecting N-glycosidase ribosome-inactivating toxins asks only “Could this sequence encode a protein capable of depurinating ribosomal RNA?” It needs only to output whether the sequence can be confidently excluded from performing the target function, whether it likely encodes the target function, or whether there is insufficient confidence to rule it out, with the latter two cases triggering review. In the near term, this classification or rule-out decision can potentially be served by small, lightweight classifiers trained on positive examples (known sequences with the target function, plus computationally generated variants) and negative examples (diverse sequences known not to have the target function), using sequence features or model embeddings as inputs. Small, specialized models can be rigorously validated against ground truth backed by experimental data and, importantly, their failure modes can be more readily characterized and understood.

The progression from specialized to generalized models is also motivated by practical considerations, as the sensitivity-specificity tradeoff may scale poorly across a large collection of independent models. An intermediate approach could use an ensemble of models that predict different molecular properties, producing a profile that can identify harmful functions. The resulting signal serves as an indicator of biosecurity risk that must be integrated into existing screening workflows where flagged sequences require expert review, making it critical to minimize false positive rates while maintaining high sensitivity.

## Tractable targets should be prioritized first

Given that “function” is a broad and ill-defined concept, we propose approaching function-based screening by prioritizing a few narrow, well-defined and highly tractable functional categories, and bootstrapping into a more generalized screening paradigm. Protein cytotoxins represent the clearest starting point: the relationship between structure and function is comparatively well-understood, decades of toxicology research provide structure-activity relationships and characterized variants, the mechanistic space is bounded, and detection aligns with current regulation of controlled toxins (CDC & USDA, 2025). Viral entry proteins, particularly receptor-binding proteins for pandemic-capable viruses, would be a natural next step. Work here can leverage advances in structure and binding affinity prediction.

More complex and context-dependent functions, such as innate immune subverting sequences and elements of fungal and protozoan pathogenesis, should be deferred due to greater challenges in data availability, context dependence, identification of host-exploiting functions, and ontological definition (Godbold et al., 2022; Godbold and Scholz, 2024). Starting with the most tractable targets and demonstrating operational feasibility builds the methodology, data pipelines, validation methods, and institutional capacity needed to expand towards more generalized function-based screening.

## Moving from concept through development to deployment

Developing function-based screening models for reliably detecting functions of concern requires several types of data: (a) positive examples, including experimentally measured natural sequences or computationally generated variants that encode the target function; (b) negative examples, including diverse sequences from organisms without the target function; and (c) held-out validation sets drawn from different taxonomic groups with little sequence or structural similarity and including experimentally validated synthetic sequences. Generating adequate high-quality training data is a significant undertaking, likely requiring several iterative rounds of variant design, data curation, model building, and validation.

It is important to acknowledge that data and modeling relating to hazardous functions are inherently sensitive, and that pursuit of such work outside of appropriately secured institutions could itself pose biosecurity risks. However, not all targets present equal sensitivity concerns. Initial development efforts should prioritize well-characterized functions of concern (e.g., well-known protein toxins) whose sequences, structures, and functional properties are already extensively documented in the open literature. For these targets, the marginal information hazard from generating additional functional variants is minimal, as the underlying biology is already widely accessible. Beginning with such targets (and employing benign proxies when possible) allows the research community to validate the full model-development pipeline while producing models with immediate defensive value. The focus should be on collecting data that accelerates defensive capabilities without generating new functional insights beyond what is necessary to advance screening.

As development progresses to less-characterized, less-public, or higher-risk functions of concern, training data becomes increasingly sensitive, as detailed information about which sequence modifications preserve toxic or pathogenic functions could itself pose a biosecurity risk. Therefore, sensitive data and trained models should be carefully controlled and distributed only through a tiered access framework (Bloomfield et al., 2026; Carter and Butchello, 2026; Wittmann et al., 2025), wherein model developers securely access the data, trusted providers and screening tool developers receive model weights for deployment, and others access screening via software-as-a-service to limit data and weight proliferation. A provider deploying a toxin-detection model can screen incoming orders without ever seeing the specific variants in the training set, keeping the information hazard contained. This framework should be implemented early on while the stakes are lower, in a graduated approach that allows operational security measures to mature and scale with the actual information hazard.

This motivates an ecosystem organized around complementary roles, in which no single entity needs access to all sensitive components. Secure research institutions generate training data, train and validate models, and conduct red-team evaluations. Trusted national and international bodies manage controlled access to models and sensitive test sets. Screening tool developers integrate validated models into production screening software, while synthesis providers deploy them and report anonymized hit patterns. And government agencies provide coordination, oversight, and threat-informed prioritization. The ecosystem should support continual improvement through operational feedback. Hit pattern reporting, expert review of flagged sequences, and emerging threat intelligence can drive rapid retraining and redeployment of individual models, creating a defense posture whose decision boundaries shift as models are updated, making them difficult to evade (Wyschogrod et al., 2022).

## Open challenges and research priorities

We believe that the approach described above is achievable with current methods and institutional capacity. Several challenges, however, will shape how quickly and successfully function-based screening can be implemented.

First, operationalizing function-based screening at any level requires clear rules for determining which biological functions warrant flagging. Several biosecurity-relevant annotation frameworks have been developed, including the Virulence Factor Database (VFDB) (Liu et al., 2022), the Pathogen–Host Interactions database (PHI-base) (Urban et al., 2022), the Functional Hazards Database (Gemler et al., 2022), Functions of Sequences of Concern (FunSoCs) (Godbold et al., 2022), PathGO (Jacak et al., 2021), and a recent formal extension of the Gene Ontology framework to pathogenic biological process terms (Godbold et al., 2025a). A key priority is to define consensus rules for determining which individual functions or combinations pose sufficient risk to warrant flagging during screening. In this regard, the recently established Sequence Biosecurity Risk Consortium (SBRC) is well positioned to develop function-based screening rubrics through careful and systematic assessment of biosecurity risk from different functions by subject matter experts (Beal and Alexanian, 2025).

Second, significant gaps remain in our understanding of where and how biological AI model predictions fail, due in part to a lack of tools and datasets to systematically evaluate their performance out of distribution. It will be crucial to develop evaluation methods and benchmarks that assess prediction accuracy for functional properties (Notin et al., 2023) for both natural and non-natural sequences across a range of protein types (Challacombe and Haas, 2024). Uncertainty quantification deserves particular attention: For any prediction used in screening, it will be important to estimate confidence, as this directly influences interpretation and determines the sensitivity and cost tradeoffs between false negatives and false positives. Robustness under adversarial conditions must also be systematically tested using structured red-teaming exercises, including whether models can maintain performance when sequences are deliberately designed to evade detection or significantly deviate from model training data (Batalis et al., 2024; Wittmann et al., 2025). Short sequence fragments carry less information and thus pose a notable challenge, as do multi-element constructs that combine coding sequences with regulatory and translational components. These challenges motivate use of mitigations such as analyzing order pools to predict plausible assembly products (Tayouri et al., 2025; Wittmann et al., 2026).

Third, there is a need to expand data collection efforts to enable training of screening-relevant models. Existing experimental data on protein function is overwhelmingly from naturally evolved sequences or close mutants, biased by common measurement techniques and functions that are easily measured in high throughput (Schnoes et al., 2013), and concentrated on a small number of model organisms (Kustatscher et al., 2022; Rocha et al., 2023). How much of functional protein space has been explored remains unclear, given evolution’s reliance on incremental sampling through mutation under selection (Hoarfrost et al., 2022; Mahlich et al., 2023). This potentially leaves vast regions uncharacterized, and hinders model training and validation. Continued progress will require scaling up experimental data collection across a wide range of protein functions (Cortade et al., 2024), with particular emphasis on characterizing more distant regions of sequence space that have not been explored by nature but are becoming accessible to biodesign. Investigating pathogenic functions poses additional challenges, as the complexity of pathogen-host interactions limits the utility of reductionist approaches (Moxon and Tang, 2000), while testing modified pathogens can pose biosecurity risks. These risks should be mitigated by using non-replicating or non-infectious models (Godbold et al., 2023) and, when possible, safe proxy functions (Ikonomova et al., 2025). Finally, functional assays have been notoriously difficult to standardize across laboratories (Hirsch and Schildknecht, 2019), and ongoing standardization efforts (Sansone et al., 2019) will be essential for reliable model development.

## Conclusion

Function-based screening provides key advantages over the current sequence-based paradigm, and is both theoretically feasible and practically achievable. Near-term priorities include defining function-based screening criteria for an initial set of targets, collecting training data on natural and computationally predicted variants, developing detection-focused models within appropriately secured research institutions, integrating function-based methods into screening tools, and piloting deployment with synthesis providers. In parallel, continued work on ontological frameworks, evaluation benchmarks, and experimental data collection across functional space will lay the groundwork for broader function-based screening. Progress along this continuum need not wait for any single challenge to be fully resolved; the near-term strategy builds the data, methods, and institutional capacity that more ambitious approaches will require. As the threat landscape evolves, so must the defenses. Screening alone cannot address all biosecurity risks, but it remains one of the most scalable, tractable, and effective points of intervention. Advancing function-based screening will require coordination among research institutions, synthesis providers, screening tool developers, and government agencies; targeted investment in training data and evaluation infrastructure; and sustained momentum from development through deployment. Together, these efforts can materialize a defensive posture that anticipates the threat landscape rather than perpetually reacts to it.

## Data Availability

The original contributions presented in the study are included in the article/supplementary material, further inquiries can be directed to the corresponding author.

## Footnotes

1 The term 'novel' is often used loosely, but novelty is not a single axis: a novel protein may diverge from a known threat in sequence while conserving structure, or diverge in structure while conserving mechanism, or diverge in mechanism while targeting the same host pathway. Different screening methods address different axes of divergence.

2 The approaches outlined here focus on protein-coding sequences as the most tractable targets for function prediction. Other biopolymers such as functional RNAs and prion-forming proteins are important, but present distinct challenges and are therefore excluded from this discussion.

## Author disclaimer

Certain commercial scientific models and software packages are identified in this paper to foster understanding. Such identification does not imply recommendation or endorsement by the National Institute of Standards and Technology, nor does it imply that the components identified are necessarily the best available for the purpose. This document does not contain technology or technical data controlled under either U.S. International Traffic in Arms Regulation or U.S. Export Administration Regulations.
